# Effect of an Anti-*Listeria* Whey Protein-Based Edible Coating Activated with Bacteriophage on Quality Attributes and Consumer Perception of Sicilian Canestrato Fresco Cheese

**DOI:** 10.3390/foods15040689

**Published:** 2026-02-13

**Authors:** Giuliana Garofalo, Chiara Pisana, Raimondo Gaglio, Marcella Barbera, Luca Settanni, Giovanni Belvedere, Giovanni Marino, Giacomo Antonio Calandra Checco, Silvia Ruta, Margherita Caccamo, Iris Schadt, Cinzia Caggia

**Affiliations:** 1 Department of Agricultural, Food and Forest Sciences, University of Palermo, Viale delle Scienze, Bldg. 5, 90128 Palermo, Italy; giuliana.garofalo01@unipa.it (G.G.); luca.settanni@unipa.it (L.S.); 2 Department of Agriculture, Food and Environment, University of Catania, Via S. Sofia, 100, 95123 Catania, Italy; chiara.pisana@phd.unict.it (C.P.); giacomo.calandrachecco@phd.unict.it (G.A.C.C.); silvia.ruta@phd.unict.it (S.R.); cinzia.caggia@unict.it (C.C.); 3 Department of Earth and Marine Sciences, University of Palermo, Via Archirafi, 22, 90123 Palermo, Italy; marcella.barbera@unipa.it; 4 Consorzio per la Ricerca nel Settore della Filiera Lattiero-Casearia e dell’Agroalimentare (CoRFiLaC), Strada Provinciale 25, km 5,200, 97100 Ragusa, Italy; belvedere@corfilac.it (G.B.); g.marino@corfilac.it (G.M.); caccamo@corfilac.it (M.C.); schadt@corfilac.it (I.S.)

**Keywords:** traditional cheese, bacteriophage, *Listeria monocytogenes*, food safety, volatile compounds, sensory traits, consumer perception

## Abstract

This study presents the first comprehensive assessment of a bacteriophage P100-activated edible whey-protein solution (WPS) applied to the rind of Sicilian Canestrato Fresco (SCF) cheese. Beyond evaluating its anti-*Listeria* efficacy in pre- and post-packaging contamination contexts, the work investigates the coating’s effects on chemical composition, volatilome, sensory properties, and consumer responses, including willingness to pay. To assess anti-*Listeria* activity, all samples were stored at 4 °C for 30 days. Contamination was carried out either before or after coating application, depending on the specific treatment. *Listeria monocytogenes* was monitored at 0, 1, 3, 7, 15, and 30 days of refrigerated storage. The active coating reduced the pathogen from approximately 3 log CFU/g to undetectable levels (0 log CFU/g) within 3 days, whereas the untreated controls reached about 5 log CFU/g after 30 days. WPS-coated cheeses showed no significant changes in chemical composition (moisture ~33%, protein ~29%, fat ~33%) or fatty acid profile compared to traditional SCF. The volatilome was dominated by hexanoic and butanoic acids and ethyl esters, without significant differences between coated and control samples, as confirmed by Smart Nose^®^ analysis. Sensory evaluation by trained assessors demonstrated that the bioactive coating did not alter the traditional sensory profile of SCF cheese. A consumer survey conducted with 240 participants from two retail formats revealed significant differences in product familiarity and perceived food safety, while openness to innovation and willingness to pay were similar. More than 90% of respondents were willing to pay a 10% price premium. Overall, phage-based edible coatings appear to be edible, renewable, and biodegradable packaging alternative to improve cheese safety without compromising quality.

## 1. Introduction

Sicilian Canestrato Fresco (SCF) is a traditional semi-hard Italian raw cow’s milk cheese and officially listed as a Traditional Agri-food Product (TAP) by the Italian Ministry of Agriculture, Food Sovereignty and Forestry [1]. Its production occurs throughout Sicily in small-scale enterprises that follow a traditional production protocol [2]. Like many historical cheeses, SCF is currently experiencing renewed consumer interest, mainly driven by its delicate flavour and versatile culinary applications [3]. Currently, after a 20-day ripening period, the cheese is vacuum-sealed using transparent multilayer films composed of petrochemical-based polymers, including polyesters, polyester amides, and polyvinyl alcohol [4]. These materials are widely available, cost-effective, and provide desirable physical properties, including heat sealability, structural strength, and effective barrier performance against gases and volatile compounds. Although these characteristics help minimize post-processing contamination and maintain product safety and quality, such packaging is neither recyclable nor biodegradable [5].

In the current era of pursuing renewable, and biodegradable solutions, both academic and industrial sectors are intensifying efforts to develop eco-friendly alternatives for dairy product packaging [6]. This approach is consistent with the objectives of the National Recovery and Resilience Plan. It also aligns with the principles of the European Green Deal, which promote nature-based solutions to support a clean, circular economy and protect public health [7,8]. In this context, the adoption of edible bioactive packaging designed to preserve both microbiological quality and sensory traits of cheese represents a promising and innovative strategy. The most proposed bioactive packaging solutions are derived from renewable sources, such as lipids, polysaccharides, and proteins [9] that can be functionalized with natural antimicrobial agents [10]. Among such agents, bacteriophages, viruses that specifically infect target bacteria without affecting other microorganisms, have recently gained prominence for their safety and effectiveness [11]. Combined with biopolymeric matrices such as whey protein phages contribute to packaging solutions for offering excellent film-forming properties, and cost-effective production [12]. Beyond these technological advantages, bacteriophages offer additional benefits compared with other natural antimicrobials commonly incorporated into edible coatings, such as essential oils, plant extracts, organic acids, or bacteriocins. Several of these compounds are known to modify the sensory attributes of cheeses [13,14]. Bacteriophages instead act with high specificity, targeting pathogenic bacteria without disturbing the autochthonous microbiota that contributes to the distinctive sensory profile of cheeses [15]. Their ability to replicate in the presence of the target pathogen also provides sustained antimicrobial activity during storage. This makes phages a particularly suitable option for improving product safety [16]. Understanding the microbiological characteristics of traditional SCF cheese is essential for assessing the relevance of phage-based packaging strategies. Such knowledge also helps evaluate their potential impact on the product.

Recently, SCF cheeses produced in different dairy factories, following traditional methods such as the use of raw cow’s milk, wooden equipment, and without any starter cultures, were evaluated from a microbiological perspective [17]. The authors reported that *Listeria monocytogenes* was not detected either in the core or the under-rind sections of all analysed samples. To our knowledge, no studies have investigated the presence of *L. monocytogenes* on the rind of SCF cheese. This surface could act as a source of pathogen dissemination during commercial distribution and consumption [18]. *L. monocytogenes* has been linked to numerous foodborne outbreaks worldwide [19]. Owing to its resilience to high salinity, low temperatures, and osmotic stress, it also poses a potential risk for cross- and post-processing contamination on the cheese rind [20]. In addition, no previous work has examined the use of phage-activated whey-protein edible coatings as an antimicrobial strategy for SCF cheese or evaluated their effects on product quality. This absence of integrated microbiological and quality focused research highlights a critical gap when considering bioactive coatings for traditional raw milk cheeses.

This research is part of a broader project primarily aimed at developing eco-friendly packaging solutions activated with bacteriophages to mitigate the risk of post-processing contamination of SCF cheeses by *L. monocytogenes*. The choice of SCF as the cheese model is justified by its short ripening period, during which the rind has not yet developed the harsh physicochemical conditions characteristic of long aged cheeses. Consequently, the SCF rind offers a more permissive environment for post-processing contamination. This makes SCF an appropriate and realistic system for evaluating both the behaviour of *L. monocytogenes* and the effectiveness of bacteriophage activated whey protein coatings. Accordingly, the research pursued two main objectives: (i) to investigate the anti-*Listeria* effectiveness of an edible bioactive whey protein-based system activated with bacteriophages on intentionally contaminated SCF cheese; and (ii) to evaluate the impact of this natural active packaging on the cheeses’ chemical composition, sensory properties assessed through both panel testing and artificial sensing, as well as consumer perception and acceptance of the final product.

## 2. Materials and Methods

### 2.1. Phage, Target Bacteria, and Raw Materials

The bacteriophage P100, targeting *L. monocytogenes*, was supplied as the commercial formulation PhageGuard Listex™ (Micreos Food Safety, Wageningen, The Netherlands). The strain *L. monocytogenes* ATCC 7644, sourced from the American Type Culture Collection and maintained at the Laboratory of Food Microbiology from the University of Palermo, was used as the target organism for sensitivity testing. Whey protein concentrate (Simplesse^®^ 100; CP Kelco, Tate & Lyle, London, UK) was used as a polymeric component for the formulation of bioactive packaging films. Glycerol (Farmalabor, Canosa di Puglia, Italy) served as a plasticizing agent, and carboxymethylcellulose (Farmalabor) was used to enhance the viscosity of the film-forming solutions, thereby improving their adhesion to the cheese rind.

### 2.2. Production of Edible Bioactive Packaging Solution

**Figure 1:**
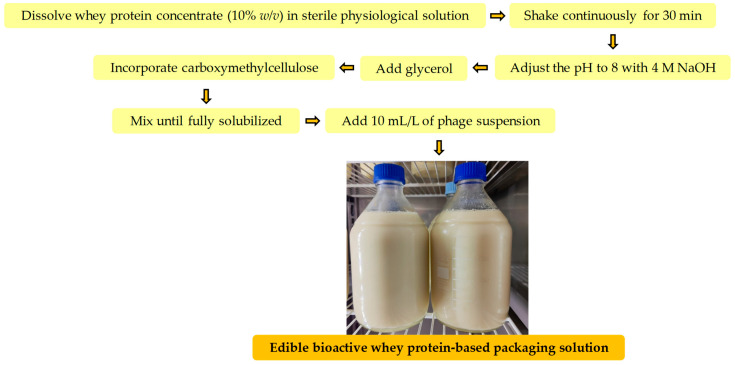
Flowchart of edible bioactive whey protein-based packaging solution production.

The whey protein-based packaging solution (WPS) was prepared following the methodologies described by Vonasek et al. [21] and García-Anaya et al. [22], with slight modifications as outlined in Figure 1.

In brief, whey protein concentrate (10% *w*/*v*) was dissolved in sterile physiological solution (Fresenius Kabi, Bad Homburg vor der Höhe, Germany) under continuous stirring for 30 min. After complete dissolution, the pH was adjusted to 8 using 4 M NaOH to promote protein denaturation. Glycerol was then added to obtain a protein-to-glycerol ratio of 2:1. Finally, carboxymethylcellulose was incorporated at 1% (*w*/*v*) and mixed until fully solubilized. The WPS was then activated by incorporating the bacteriophage under sterile conditions within a laminar flow hood at room temperature. Specifically, 10 mL/L of phage suspension solution was added, resulting in a final concentration between 1 × 10^10^ and 1 × 10^11^ plaque-forming units (PFU)/mL, as confirmed by direct plating plaque assay using the *L. monocytogenes* ATCC 7644 strain.

### 2.3. Enumeration and Detection of L. monocytogenes on Edible Bioactive Packaging Solution and Cheese Rind

The enumeration and detection of *L. monocytogenes* on WPS and SCF cheese rind were performed in accordance with the International Organization for Standardization (ISO) guidelines. Specifically, enumeration followed the ISO 11290-2 [23], involving serial dilutions in Maximum Recovery Diluent (MRD; Liofilchem^®^, Roseto degli Abruzzi, Italy). For WPS samples, 1 mL was diluted directly, whereas approximately 10 g of cheese rind (≈0.5 cm thick) was aseptically collected with a sterile scalpel and homogenized in 90 mL of MRD before dilution. Subsequently, 100 µL aliquots from each dilution were plated onto agar *Listeria* according to Ottaviani and Agosti (ALOA; Biolife Italiana, Monza, Italy) and incubated at 37 °C for 24 h. The detection of *L. monocytogenes* was carried out according to ISO 11290-1 [24] on 25 mL of WPS and 25 g of cheese rind, following a two-step enrichment in Half Fraser and Fraser broth media (VWR International, Darmstadt, Germany). The quantification limit of the enumeration method [23] was <1 CFU/mL for liquid samples (WPS) and <2 log CFU/g for solid samples (cheese rind). In contrast, the qualitative detection assays [24] had a detection limit equivalent to 0 log CFU/mL or g for both liquid and solid matrices. Analyses were performed in duplicate.

### 2.4. In Vivo Determination of Anti-Listeria Activity

#### 2.4.1. Preparation of *L. monocytogenes* Culture for Challenging Test

The reference strain *L. monocytogenes* ATCC 7644 was sub-cultured in Brain Heart Infusion (BHI; Lickson S.r.l., Vicari, Italy) broth and incubated for 1 day at 37 °C. After incubation, the cells were harvested by centrifugation at 10,000× *g* for 5 min. They were then washed twice with MRD and re-suspended in the same solution. The suspension was adjusted to an optical density at 600 nm (OD_600_) of approximately 1.00, measured using a DR 3900 Spectrophotometer (Hach-Lange Ltd., Manchester, UK). This OD corresponded to an estimated concentration of 10^9^ CFU/mL, as confirmed by plate counting. The resulting suspension was then applied to the SCF cheese rinds at a final cell density of about 10^4^ CFU/g to simulate a severe contamination.

#### 2.4.2. Experimental Plan

To evaluate the anti-*Listeria* effect of WPS on SCF cheese rinds, *in vivo* trials were performed at the dairy pilot plant of the CoRFiLaC (Consorzio per la Ricerca nel Settore della Filiera Lattiero-casearia e dell’Agroalimentare, Ragusa, Italy). The experimental design included five independent trials: two under uncontaminated conditions (CPU and EPU) and three intentionally contaminated with *L. monocytogenes* ATCC 7644 (CPC, EPC-A, and EPC-B), as summarized in Table 1.

**Figure 2:**
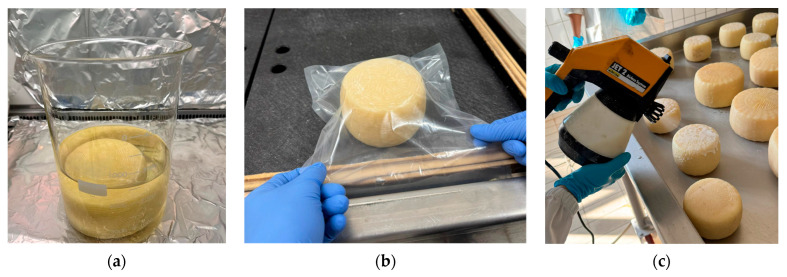
*In vivo* contamination test. (**a**) cheese contamination; (**b**) control production contaminated with *L. monocytogenes* ATCC 7644, vacuum-sealed in conventional non-biodegradable plastic bags; (**c**) coating application of edible bioactive whey protein-based solution.

The three contaminated trials were designed to simulate the most plausible real-world contamination pathways that may occur during cheese production and commercialisation. In all trials, whole 1 kg wheels of traditional SCF cheese were immersed in a *L. monocytogenes* ATCC 7644 cell suspension to obtain a final concentration of approximately 10^3^ CFU/mL (Figure 2a). For the control production (CPC), the contaminated wheels were vacuum-packed in non-biodegradable plastic bags (Figure 2b), reproducing current industrial practices. In the experimental treatment EPC-B, the cheese wheels were coated with the WPS after contamination (Figure 2c). This approach simulates pre-packaging contamination events that may occur during cheese handling, manipulation, or storage in small scale dairies.

In contrast, EPC-A samples were contaminated after the application and drying of the coating, thereby simulating post-packaging contamination that may arise during retail handling, slicing, or consumer manipulation. After the application of the WPS solution, EPC-B cheeses underwent a drying phase at room temperature for 45 min, which was necessary to promote the adhesion of the edible coating to the product. In contrast, for EPC-A cheeses, this drying phase was carried out before the contamination with *L. monocytogenes*. Uncontaminated control (CPU) and experimental (EPU) productions were also included in the study. The CPU samples were vacuum-packed in non-biodegradable plastic bags, whereas the EPU samples were coated with the WPS. These uncontaminated cheeses were used for sensory evaluations and consumer perception tests. All uncontaminated and contaminated cheese samples were separately stored in two refrigerated cabinets (Afinox srl, Marsango, Italy) at 4 °C for 30 days.

Enumeration and detection of *L. monocytogenes* in all contaminated samples were assessed at five storage times (0, 1, 3, 7, 15, and 30 days), following the procedure already described. To ensure experimental reliability, all trials were carried out three times independently. At each sampling point, microbiological determinations were performed in duplicate.

### 2.5. Analysis of Proximate and Fatty Acid Composition

The proximate composition of control (CPU) and experimental (EPU) cheese samples, including fat, moisture, protein, and sodium chloride content, was quantitatively assessed using predictive models based on mid-infrared spectroscopy (MIRS), according to the procedure outlined by Scatassa et al. [25].

The fatty acid (FA) profile was analyzed using Gas Chromatography–Mass Spectrometry (GC-MS; Agilent Technologies, Santa Clara, CA, USA). Preparation of the sample, including lipid extraction and derivatization to FA methyl esters (FAMEs), was carried out as previously described by Pisana et al. [17]. Chromatographic separation conditions and data acquisition parameters were applied as reported in a previous study [17]. FA were identified and quantified by matching their chromatographic retention times with those of a certified reference mixture (Supelco 37 Component FAME Mix, Sigma-Aldrich, St. Louis, MO, USA). All analyses were conducted in duplicate for each independent batch of production.

### 2.6. Volatile Organic Compounds Detection Through SPME-GC-MS and Smart Nose^®^

Volatile organic compounds (VOCs) were analyzed by headspace solid-phase microextraction (HS-SPME) coupled with gas chromatography–mass spectrometry (GC–MS; Agilent 5975C, Agilent Technologies, Santa Clara, CA, USA). Cheese samples (5 g) were subjected to HS-SPME using a DVB/CAR/PDMS fiber (50 mm, Supelco, Bellefonte, PA, USA), as detailed by Pisana et al. [17]. Chromatographic separation of extracted compounds and full-scan mass spectrometric acquisition were carried out following previously established analytical conditions [17]. Volatile compounds were assigned based on mass spectral matching with entries in the NIST library. Cheese samples were subsequently analyzed using an electronic nose system (SMart Nose, LDZ, CH-2074 Marin-Epagnier, Zwingen, Switzerland), which performs direct mass-spectrometric detection of VOCs without prior headspace separation. The platform consists of a Combi Pal autosampler (CTC Analytics AG, Cycle Composer software, version 1.52, Zwingen, Switzerland) coupled to a high-sensitivity quadrupole mass spectrometer (Inficon AG; detection range 1–200 amu) and controlled through SMart Nose 1.51 software for signal acquisition and multivariate data processing. For each measurement, 4 g of cheese were transferred into 10 mL glass vials compatible with the autosampler and sealed with butyl/PTFE septa and caps. Vials were randomly arranged in the autosampler tray to minimize positional bias, and three replicate injections were carried out per sample. Operating parameters were set as follows: incubation temperature 60 °C; incubation time 30 min; injection volume 2.5 mL; syringe temperature 100 °C; injector temperature 160 °C; nitrogen purge flow 200 mL/min; EI ionization energy 70 eV; scan rate 0.5 s/mass; mass range 10–160 amu; secondary electron multiplier voltage 1540 V. Each run lasted 170 s, enabling the acquisition of three complete scan cycles per injection. All analyses were conducted in duplicate for each independent batch of production.

### 2.7. Sensory Analysis

Cheese samples stored for 30 days under refrigeration were subjected to sensory evaluation using Quantitative Descriptive Analysis (QDA), in accordance with UNI EN ISO 13299 [26] guidelines. Uniform slices of approximately 10 g, which included the rind and had not undergone any washing, were equilibrate to room temperature (20 ± 2 °C) for 30 min, coded, and presented on white paperboard plates in random order. The evaluation was performed by a panel of 10 trained assessors in individual booths within the sensory laboratory of CoRFiLaC, under controlled conditions compliant with UNI EN ISO 8589 [27] standards for sensory analysis environments. Seventeen descriptors grouped into aspect (cheese rind color, rind color uniformity, cheese core color, and uniformity of structure), aroma (odor intensity, typical odor, milk odor, butter odor, and aroma intensity), taste (sweet, salt, bitter, and acid), and texture (hardness, plastic, mellow, and dispersion) categories were assessed, as reported by Caccamo et al. [28]. All attributes were rated using a 9-point hedonic scale (1 = low intensity; 9 = high intensity).

### 2.8. Consumer Perception and Acceptance Analysis

A cross-sectional survey was conducted to evaluate consumer perception and acceptance of SCF cheese packaged with the bioactive WPS solution. The study comprised 240 respondents recruited from two retail formats located in Modica (Ragusa, Italy): a premium store “Superstore Le Liccumie” (n = 125; 52%) and a convenience-oriented store Conad (n = 115; 48%). Data were collected through face-to-face interviews using a structured questionnaire. The survey covered socio-demographic characteristics, consumption habits, and perceptions of microbiological safety in cheeses. It also explored openness to active packaging innovations and purchase intentions, including willingness to pay for the new product. The procedure followed approaches described by Ares and Gámbaro [29] and Grunert et al. [30]. Table 2 presents the socio-demographic profile of the participants, including gender, age groups (18–24, 25–34, 35–44, 45–54, 55–64, 65+), education level (primary school, secondary school, university), and household size (1, 2, or ≥3 members).

After starting the investigation, participants received a comprehensive explanation of the characteristics and functions of active packaging technologies, consistent with the approach outlined by Cammarelle et al. [31].

### 2.9. Statistical Analysis

Microbiological, chemical, and sensory data were subjected to one-way ANOVA, and pairwise differences were assessed using Tukey’s post hoc procedure. VOCs emitted from cheeses were represented using a heat map created through ascending hierarchical clustering. Each VOC concentration was displayed using a color gradient ranging from yellow (lowest level) to red (highest level).

The VOCs detected by the SMart Nose^®^ system were explored through principal component analysis (PCA) to highlight the ions contributing most strongly to sample differentiation and to visualize variations in the volatile profiles of the cheeses. All statistical elaborations were performed using the software integrated in the SMart Nose^®^ platform. Although PCA is not inherently designed for classification, the software allows sample grouping based on Euclidean distances within the multidimensional space defined by the principal components. For each discrimination pattern, the parameters used to compute the component scores were adjusted accordingly.

The frequencies of responses obtained from the questionnaire, structured to evaluate the perception and acceptance of SCF cheese packaged with the active packaging were examined using the Chi-square test. Statistical significance was defined at *p* ≤ 0.05. All computations were carried out using XLStat (version 2020.3.1; Addinsoft, New York, NY, USA) implemented in Microsoft Excel.

## 3. Results and Discussion

### 3.1. Assessment of L. monocytogenes Presence on Bioactive WPS, Traditional SCF, and Experimental Cheese Rinds

**Figure 3:**
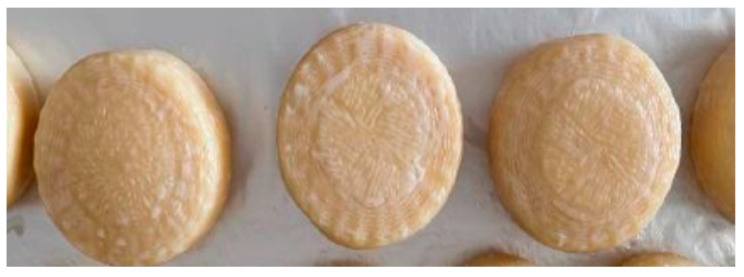
Sicilian Canestrato Fresco cheese coated with edible bioactive WPS solution.

The edible bioactive WPS solution applied as active packaging on SCF cheese rinds (Figure 3) was evaluated for its *in vivo* anti-*Listeria* activity.

**Figure 4:**
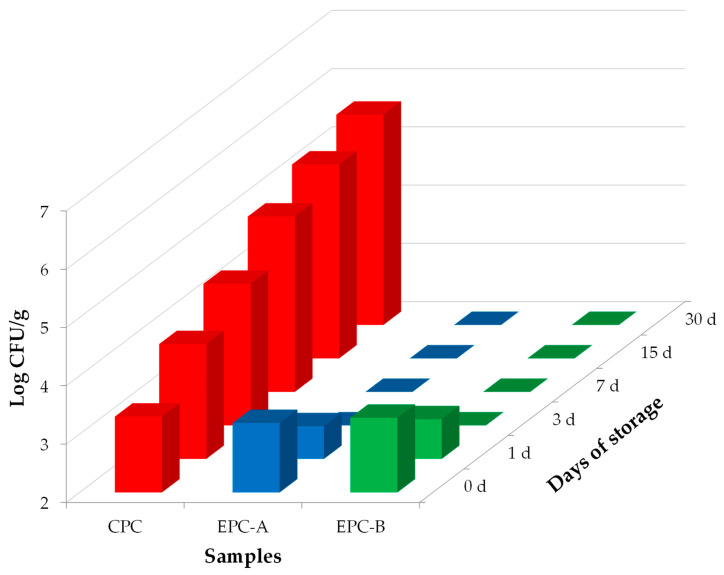
Growth trend of *Listeria monocytogenes* on control and experimental Sicilian Canestrato Fresco cheese rinds during refrigerated storage. Abbreviations: CPC, control production contaminated with *L. monocytogenes* ATCC 7644, vacuum-sealed in conventional non-biodegradable plastic bags; EPC-A, experimental production contaminated with *L. monocytogenes* ATCC 7644 after application of bioactive whey protein solution.; EPC-B, experimental production contaminated with *L. monocytogenes* ATCC 7644 before application of bioactive whey protein solution.

The results of the enumeration and detection on *L. monocytogenes* are reported in Figure 4. Data on *L. monocytogenes* in WPS and traditional SCF cheese rinds are not included, as the microorganism was not detected in any sample (0 log CFU/mL for WPS and 0 log CFU/g for SCF cheese rinds). As graphically represented at the initial sampling time (day 0), both the control (CPC) and experimental cheese samples (EPC-A and EPC-B) exhibited *L. monocytogenes* levels comparable to the initial inoculated concentration, confirming that the inoculum was performed at approximately 3 log CFU/g. In CPC *L. monocytogenes* exhibited a progressive increase of approximately 0.5 log units at each sampling point, reaching about 5 log CFU/g after 30 days of refrigerated storage.

These results align with previous studies indicating that *L. monocytogenes* can survive and proliferate on cheese rinds stored under vacuum or modified atmosphere packaging, thereby representing a significant consumer risk [32]. The ability of this pathogen to persist on cheese rinds is attributable to its intrinsic tolerance to salt, its capacity to grow under anaerobic conditions, and its ability to proliferate at refrigeration temperatures [33,34].

Conversely, in the EPC-A and EPC-B cheeses, the application of the bioactive WPS exerted a marked inhibitory effect, driving *L. monocytogenes* counts below the enumeration threshold (<2 log CFU/g) after 3 days of storage.

This finding was further confirmed by detection analyses, which did not reveal the pathogen from that time point onward (0 log CFU/g). The *in vivo* anti-*Listeria* activity observed in the coated cheeses is consistent with previous studies showing that whey-protein matrices can enable a gradual release of bacteriophages [21,35]. The behaviour recorded in our samples aligns with the phage release dynamics described in those works, which are known to vary depending on the physicochemical properties of the packaging material. In addition, the antimicrobial effectiveness of the WPS solution is primarily attributable to the high specificity and strong lytic activity of phage P100 against *L. monocytogenes* [36], as well as to the ability of whey protein matrices to protect phages from environmental stressors and ensure their gradual and sustained release over time [21]. Interestingly, this antimicrobial action remains confined to the cheese rind and does not interfere with the biochemical processes occurring in the under-rind layer or in the core of the cheese, which are essential for the development of its characteristic quality attributes [21,35]. Our results demonstrate that the WPS solution activated with the P100 phage effectively inhibits the growth and survival of *L. monocytogenes* on SCF cheese rinds. This effect occurs regardless of whether contamination takes place before or after the coating is applied. The treatment therefore enhances food safety by reducing consumer exposure to the pathogen. Importantly, the complete elimination of *L. monocytogenes* from SCF cheese rinds also has regulatory significance. It ensures compliance with the microbiological safety criteria established for raw milk dairy products by Commission Regulation (EC) No 2073 [37].

### 3.2. Proximate and Fatty Acid Composition of Cheeses

Table 3 reports the proximate chemical composition and FA profile of CPU and EPU cheese samples. No significant differences were detected between the two packaging systems, as all comparisons yielded *p*-values greater than 0.05. The moisture, protein, and fat contents were approximately 30% in both types of cheese. The distribution of FAs was consistent, with long-chain FA (LCFA, C15–C18) predominating, followed by medium-chain (MCFA, C10–C14) and short-chain FA (SCFA, C6–C10). Among the LCFAs, saturated FA (SFA) were the most abundant, with palmitic acid (C16:0) consistently representing the major component, followed by myristic acid (C14:0) and stearic acid (C18:0), all characteristic of bovine milk fat. Within the monounsaturated fraction, oleic acid (C18:1 *cis*9) was the predominant compound.

These values are in line with those typically reported for SCF cheeses [17] and other traditional Italian cheeses produced from raw cow’s milk [38,39]. They show a predominance of long-chain SFA and oleic acid, reflecting the typical composition of bovine milk fat and the limited lipolysis characteristic of fresh and semi-hard cheeses. Furthermore, the lack of variability in composition confirms that the application of the bioactive WPS solution did not affect cheese composition.

### 3.3. Cheese Volatilome

**Figure 5:**
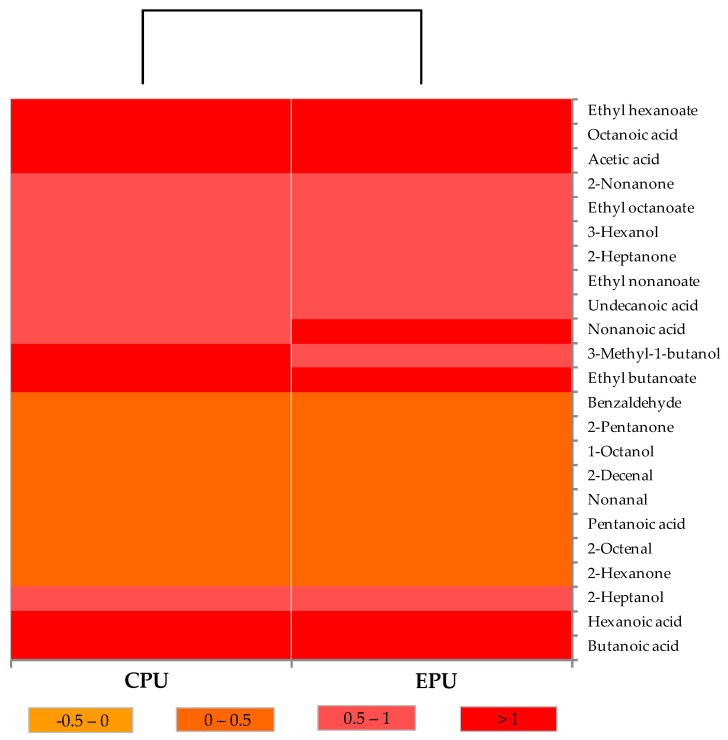
Distribution of volatile organic compounds emitted from control and experimental Sicilian Canestrato Fresco cheeses. The heat map illustrates the relative abundance of each compound. Abbreviations: CPU, control production under uncontaminated conditions, vacuum-sealed in conventional non-biodegradable plastic bags; EPU, experimental production under uncontaminated conditions, packaged with bioactive whey protein solution.

The VOC profiles of cheese samples are reported in Figure 5. The composition included organic acids, esters, alcohols, ketones, and aldehydes, consistent with profiles typically reported for SCF cheeses [17]. In both CPU and EPU cheese samples, free FA (FFA) were the dominant class, with hexanoic and butanoic acids as the most abundant, reflecting the typical profile of cow’s milk cheeses [40,41,42]. Esters, mainly formed through esterification of alcohols and FA, represented the second most abundant group and contributed fruity and floral notes; ethyl hexanoate was the most prominent, followed by ethyl butanoate, ethyl octanoate, and ethyl decanoate [43,44]. Alcohols, aldehydes, and ketones occur at lower concentrations.

**Figure 6:**
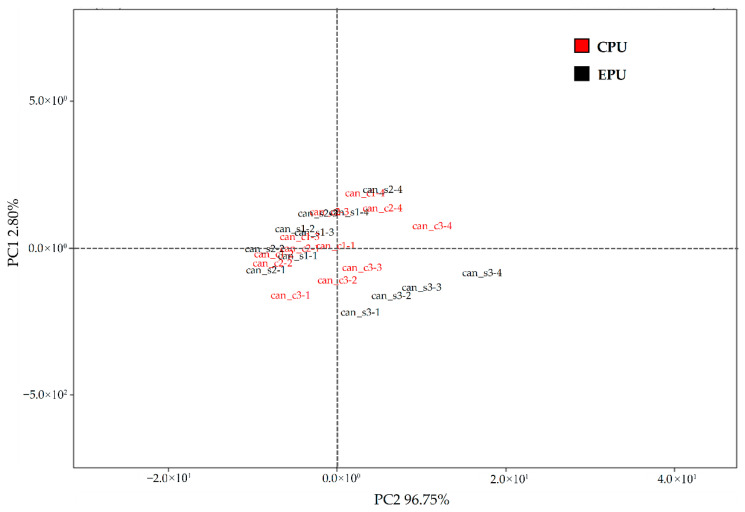
Score plot derived from the principal component analysis (PCA) of volatile organic compounds (VOCs) profiles measured with the SMart Nose^®^ system. Points represent individual replicates. PC1 and PC2 together captured 99.55% of the dataset’s variability (96.75% and 2.80%, respectively). Abbreviations: CPU, control production under uncontaminated conditions, vacuum-sealed in conventional non-biodegradable plastic bags; EPU, experimental production under uncontaminated conditions, packaged with bioactive whey protein solution.

These results were confirmed by Smart Nose analysis, which showed no statistically significant differences between CPU and EPU samples (Figure 6). Results of PCA revealed a strong overlap between the two groups, with no clear separation along the main components explaining the variance (PC1: 96.75%; PC2: 2.80%), indicating VOC profiles. Likewise, classification models commonly applied to electronic nose data (e.g., linear discriminant analysis) did not achieve meaningful discrimination between treatments, suggesting that the packaging strategy did not influence the aromatic fingerprint of the cheese.

The lack of significant variation in the aromatic fingerprint between CPU and EPU corroborates earlier findings that whey protein coatings do not appreciably influence volatile profiles [45,46], thereby maintaining the distinctive aromatic identity of SCF cheeses.

### 3.4. Sensory Evaluation of Cheeses

**Figure 7:**
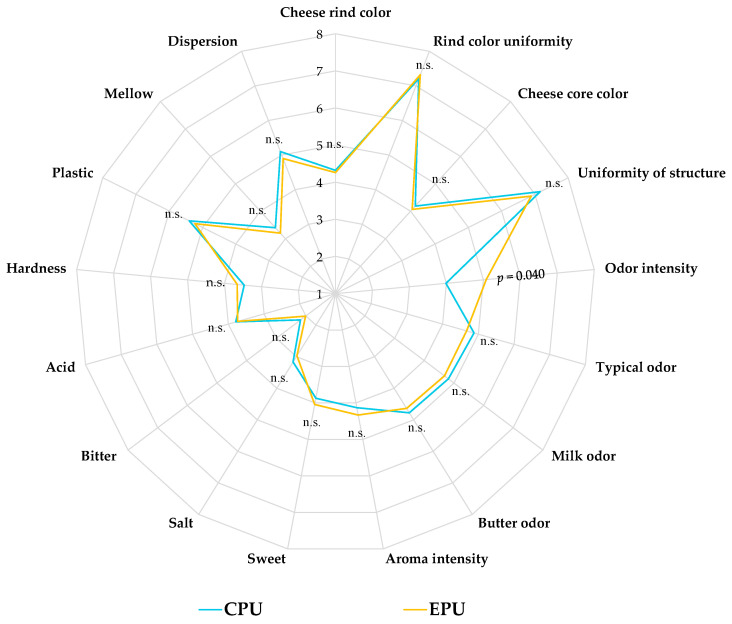
Radar chart of descriptive sensory analysis of control and experimental Sicilian Canestrato Fresco cheeses. Abbreviations: CPU, control production under uncontaminated conditions, vacuum-sealed in conventional non-biodegradable plastic bags; EPU, experimental production under uncontaminated conditions, packaged with bioactive whey protein solution; n.s., not significant.

The radar chart depicting the sensory attributes of CPU and EPU cheese samples after 30 days of refrigerated storage, as assessed during tasting sessions, is shown in Figure 7.

This evaluation is essential to confirm that edible packaging preserves appearance and aroma traits, which are key drivers of consumer acceptance and product authenticity. Consequently, ensuring that innovative edible films maintain these attributes is critical for successful market adoption [47]. In this study, the application of bioactive WPS as an edible packaging solution did not significantly affect the sensory profile of SCF cheeses. Except for odour intensity, all other evaluated attributes showed no significant statistical differences following WPS application. In particular, the experimental cheese packaged with bioactive WPS exhibited higher odour intensity values. Furthermore, the absence of substantial differences between control and experimental samples for most sensory attributes can likely be attributed to the use of the same raw cow’s milk in both cases. Indeed, cheese sensory characteristics are primarily shaped by intrinsic and extrinsic factors, including the microbiological status of the milk and farming practices such as animal diet and herd management [48].

Overall, these findings indicate that the application of the WPS solution as a packaging system for SCF cheese does not alter the product’s sensory characteristics.

### 3.5. Assessment of Consumer Perceptions and Acceptance

Consumer perceptions and acceptance of SCF cheese packaged with the bioactive WPS solution were assessed through a survey involving 240 participants recruited from two retail formats representing distinct store classifications. The selection of different retail environments aimed to capture differences in consumer profiles between a premium store and a convenience-oriented supermarket, an approach consistent with previous research on consumer segmentation in novel food technology acceptance [49,50]. Such evaluation is considered essential before introducing a new food product into the market to ensure its acceptability among consumers [51]. In fact, even when an innovation is technologically effective, its market uptake ultimately depends on consumers’ willingness to purchase foods treated with such technologies [52]. As reported in Table 2, respondents at Superstore Le Liccumie were predominantly female, older (55–64 years), and characterized by higher levels of schooling, whereas those at Conad Modica were mostly male, slightly younger, and with lower level of school education. These socio-demographic distinctions suggest that the premium store attracts a more informed, quality-oriented clientele, while the convenience-oriented store serves a pragmatic, price-sensitive segment. These findings are consistent with those reported by Gagliardi et al. [53], who emphasize that education level plays a crucial role in shaping consumer attitudes toward food products. The results for all variables included in the questionnaire are presented in Table 4.

Statistical differences emerged between the two retail formats with respect to familiarity with traditional SCF cheese (χ^2^ = 11.24, *p* = 0.004) and perceived food safety (χ^2^ = 11.79, *p* = 0.003). In contrast, no significant variation was observed in consumer attitudes toward innovation (χ^2^ = 2.06, *p* = 0.357) or in their willingness to pay a premium for the innovative SCF cheese packaged with an anti-*Listeria* WPS solution (χ^2^ = 0.01, *p* = 0.906). Purchasing patterns of traditional SCF cheese vary across retail formats. In the Superstore, more than half of respondents (51.8%) reported familiarity with the product, yet only 8.2% purchased it regularly, indicating informed but lower exploratory behaviour. At Conad, overall awareness was lower, but regular purchase was higher (21.2%), despite 47.2% of consumers declaring no knowledge of the product. This suggests a more habitual consumer base with potential for local loyalty. Comparable results have been reported in studies on traditional cheeses, where awareness often enhances perceived value but does not necessarily lead to frequent consumption [49]. Perceptions of food safety were generally high, but with notable variation between formats: 91.8% of Superstore customers felt “very” or “moderately safe” purchasing traditional cheese, compared to 78.9% at Conad. The higher confidence observed at the Superstore likely reflects its premium positioning and perceived control over quality, reinforcing the role of retail environment in shaping trust [54]. Openness to innovation was elevated across retail contexts, with 58.7% of respondents at the Superstore and 63.2% at Conad expressing favourability toward purchasing cheese with active packaging. This result aligns with previous research on consumer acceptance of food technologies aimed at improving the safety of dairy products through natural preservation methods [55]. These studies highlight a growing preference for solutions that extend shelf life while preserving product authenticity. They also show increasing consumer interest in approaches that minimize the use of synthetic preservatives. Purchase intention and willingness to pay further corroborated the positive reception of the innovation. Over 90% of respondents in both retail formats expressed readiness to pay a 10% price premium for the new product. These results reinforce evidence that perceived safety and quality frequently outweigh price sensitivity in traditional food markets [56]. Overall, these findings show that bioactive packaging for SCF cheese is well accepted across different retail settings. This broad appeal reinforces its potential as a safety enhancing innovation in traditional food markets.

## 4. Conclusions

This study provides the first integrated evaluation of a whey protein–based edible coating activated with bacteriophage P100 for the protection of SCF. The bioactive WPS solution completely eliminated *L. monocytogenes*, reducing initial contamination levels of approximately 3 log CFU/g to 0 log CFU/g within 3 days of refrigerated storage. The WPS application did not modify the cheese’s proximate composition, its FA profile, and the volatilome remained dominated by hexanoic and butanoic acids and related ethyl esters. Sensory evaluation confirmed the preservation of the traditional attributes of SCF cheese, and consumer testing revealed strong acceptance and high willingness to pay for the innovative product. Compared with other edible coatings incorporating essential oils, plant extracts, or organic acids, the phage-based system offers the advantage of highly targeted antimicrobial activity without altering sensory characteristics. In addition, unlike conventional plastic vacuum packaging, this whey protein matrix represents a renewable and biodegradable solution aligned with current national and European sustainability objectives. Ongoing research is focused on validating the performance of this bioactive solution across different traditional cheese typologies. It also examines how the solution behaves along commercial distribution chains. In addition, researchers are exploring phage stability and release dynamics. These efforts aim to further strengthen the technological applicability of the solution.

## Figures and Tables

**Table 1:** Experimental design.

Trials	Type of Material	Packaging	Contamination
CPU	plastic bags	under vacuum	-
EPU	whey protein solution	coating	-
CPC	plastic bags	under vacuum	Before packaging
EPC-A	whey protein solution	coating	After application of the whey protein solution
EPC-B	whey protein solution	coating	Before application of the whey protein solution

Abbreviations: CPU, control production under uncontaminated conditions vacuum-sealed in conventional non-biodegradable plastic bags; EPU, experimental production under uncontaminated conditions, packaged with bioactive whey protein solution; CPC, control production contaminated with *L. monocytogenes* ATCC 7644, vacuum-sealed in conventional non-biodegradable plastic bags; EPC-A, experimental production contaminated with *L. monocytogenes* ATCC 7644 after application of bioactive whey protein solution.; EPC-B, experimental production contaminated with *L. monocytogenes* ATCC 7644 before application of bioactive whey protein solution. Symbol: - no contamination.

**Table 2:** Socio-demographic details of the analysed consumer sample (% per retail format).

Characteristic	Superstore Le Liccumie		Conad Modica	
	Total (*n*)	%	Total (*n*)	%
Gender				
Female	73	57.9	48	42.1
Male	52	42.1	67	57.9
Age				
18–24	13	10.0	13	11.2
25–34	22	17.9	19	16.7
35–44	18	14.6	19	16.7
45–54	21	17.1	32	27.9
55–64	43	34.1	26	22.1
65+	8	6.3	6	5.4
Education				
Primary school	32	25.8	6	5.4
Secondary school	42	33.3	85	73.8
University	51	40.9	24	20.8
Household dimension				
1	23	18.3	18	15.8
2	20	16.3	37	31.7
3 or more	82	65.4	60	52.5

**Table 3:** Proximate composition (%) and fatty acids profiles (g/100 g FA) of control and experimental Sicilian Canestrato Fresco cheeses.

Parameters	Samples		SEM	*p*-Value
	CPU	EPU		
Moisture	34.61	32.86	0.230	0.125
Protein	28.01	29.16	0.156	0.144
Fat	32.45	33.81	0.201	0.191
NaCl	0.88	0.92	0.013	0.597
Caproic acid (C6:0)	2.41	2.35	0.019	0.573
Caprylic acid (C8:0)	1.63	1.54	0.014	0.240
Capric acid (C10:0)	3.73	3.40	0.040	0.088
Lauric acid (C12:0)	4.41	4.09	0.042	0.129
Myristic acid (C14:0)	12.80	12.20	0.106	0.291
Pentadecanoic acid (C15:0)	1.46	1.53	0.013	0.318
Palmitic acid (C16:0)	34.38	32.29	0.242	0.063
Palmitoleic acid (C16:1)	1.97	1.85	0.018	0.197
Stearic acid (C18:0)	11.76	11.63	0.088	0.799
Oleic acid (C18:1 cis)	20.54	21.17	0.166	0.501
Oleic acid (C18:1 trans)	1.50	1.61	0.016	0.167
Linoleic acid (C18:2)	2.81	2.66	0.024	0.245

Results indicate mean values six determinations (performed in duplicate for three independent productions). Abbreviations: CPU, control production under uncontaminated conditions, vacuum-sealed in conventional non-biodegradable plastic bags; EPU, experimental production under uncontaminated conditions, packaged with bioactive whey protein solution; SEM, standard error of mean.

**Table 4:** Determinants of consumer perception and acceptance across retail formats.

Parameters	Retail Formats		χ^2^	*p*-Value
	Superstore Le Liccumie	Conad Modica		
Familiarity with traditional SCF cheese				
Regular purchase	8.2	21.2	11.24	0.004
Known but purchased rarely	51.8	31.6		
Not known	40	47.2		
Perceived food safety				
Very safe	64.5	42.1	11.79	0.003
Moderately safe	27.3	36.8		
Slightly safe	8.2	21.1		
Openness to innovation				
High openness	58.7	63.2	2.06	0.357
Conditional openness with clear explanation	39.5	36.8		
No openness	1.8	0		
Willingness to pay				
Affirmative	93.2	94.6	0.01	0.906
Negative	6.8	5.4		

Abbreviations: SCF, Sicilian Canestrato Fresco.

## Data Availability

All primary data generated in this study are provided within the article. Any additional information can be requested from the corresponding author.

## References

[1] Gazzetta Ufficiale della Repubblica Italiana Decreto Ministeriale 29 Febbraio 2024: Aggiornamento dell’Elenco Nazionale dei Prodotti Agroalimentari Tradizionali ai sensi dell’Art. 12, Comma 1, della Legge 12 Dicembre 2016, n. 238 (24A01305). GU Serie Generale 2024, 61. https://www.gazzettaufficiale.it/eli/id/2024/03/13/24A01305/SG.

[2] Regione Siciliana. Assessorato dell’Agricoltura e delle Foreste (1999). Decreto 28 dicembre 1998. Riconoscimento di prodotti a base di latte come prodotti storici fabbricati tradizionalmente. Gazz. Uff. Reg. Sicil..

[3] Assolatte (2022). Formaggi Freschi Italiani: Primosale.

[4] Piscopo A., Zappia A., De Bruno A., Pozzo S., Limbo S., Piergiovanni L., Poiana M. (2019). Use of biodegradable materials as alternative packaging of typical Calabrian Provola cheese. Food Packag. Shelf Life.

[5] Siracusa V., Rocculi P., Romani S., Rosa M.D. (2008). Biodegradable polymers for food packaging: A review. Trends Food Sci. Technol..

[6] Siciliano S., Lopresto C.G., Lamonaca F. (2024). From traditional packaging to smart bio-packaging for food safety: A review. Euro-Mediterr. J. Environ. Integr..

[7] Pilati M. (2021). National Recovery and Resilience Plans: Empowering the Green and Digital Transitions?.

[8] Toth G. (2019). Circular economy and its comparison with 14 other business sustainability movements. Resources.

[9] Gamage A., Thiviya P., Liyanapathiranage A., Wasana M.L.D., Jayakodi Y., Bandara A., Manamperi A., Dassanayake R.S., Evon P., Merah O. (2024). Polysaccharide-Based Bioplastics: Eco-Friendly and Sustainable Solutions for Packaging. J. Compos. Sci..

[10] Moustafa H., Youssef A.M., Darwish N.A., Abou-Kandil A.I. (2019). Eco-friendly polymer composites for green packaging: Future vision and challenges. Compos. Part B Eng..

[11] Lone A., Anany H., Hakeem M., Aguis L., Avdjian A.C., Bouget M., Griffiths M.W. (2016). Development of prototypes of bioactive packaging materials based on immobilized bacteriophages for control of growth of bacterial pathogens in foods. Int. J. Food Microbiol..

[12] Wagh R.V., Priyadarshi R., Khan A., Riahi Z., Packialakshmi J.S., Kumar P., Rhim J.W. (2025). The role of active packaging in the defense against foodborne pathogens with particular attention to bacteriophages. Microorganisms.

[13] Busetta G., Ponte M., Barbera M., Alfonzo A., Ioppolo A., Maniaci G., Guarcello R., Francesca N., Palazzolo E., Bonanno A. (2022). Influence of citrus essential oils on the microbiological, physicochemical and antioxidant properties of Primosale cheese. Antioxidants.

[14] Garofalo G., Ponte M., Greco C., Barbera M., Mammano M.M., Fascella G., Greco G., Salsi G., Orlando S., Alfonzo A. (2023). Improvement of fresh ovine “Tuma” cheese quality characteristics by application of oregano essential oils. Antioxidants.

[15] Grigore-Gurgu L., Bucur F.I., Mihalache O.A., Nicolau A.I. (2024). Comprehensive review on the biocontrol of *Listeria monocytogenes* in food products. Foods.

[16] Hoffmann A., Sadowska K., Zenelt W., Krawczyk K. (2025). Post-Harvest Disease Control Using Bacteriophages: Current Strategies, Practical Applications, and Future Trends. Agriculture.

[17] Pisana C., Caccamo M., Barbera M., Marino G., Serio G., Franciosi E., Settanni L., Gaglio R., Caggia C. (2025). Comprehensive characterization of the microbiological and quality attributes of traditional Sicilian Canestrato fresco cheese. Foods.

[18] Gérard A., El-Hajjaji S., Niyonzima E., Daube G., Sindic M. (2018). Prevalence and survival of *Listeria monocytogenes* in various types of cheese—A review. Int. J. Dairy Technol..

[19] Shamloo E., Hosseini H., Moghadam Z.A., Larsen M.H., Haslberger A., Alebouyeh M. (2019). Importance of *Listeria monocytogenes* in food safety: A review of its prevalence, detection, and antibiotic resistance. Iran. J. Vet. Res..

[20] Schneider G., Steinbach A., Putics Á., Solti-Hodován Á., Palkovics T. (2023). Potential of essential oils in the control of *Listeria monocytogenes*. Microorganisms.

[21] Vonasek E., Le P., Nitin N. (2014). Encapsulation of bacteriophages in whey protein films for extended storage and release. Food Hydrocoll..

[22] García-Anaya M.C., Sepulveda D.R., Rios-Velasco C., Acosta-Muñiz C.H. (2023). Incorporation of A511 Bacteriophage in a Whey Protein Isolate-Based Edible Coating for the Control of *Listeria monocytogenes* in Cheese. Food Packag. Shelf Life.

[23] (2017). Microbiology of Food Chain—Horizontal Method for the Detection and Enumeration of *Listeria monocytogenes* and of *Listeria* spp.—Part 2: Enumeration Method.

[24] (2017). Microbiology of the Food Chain—Horizontal Method for the Detection and Enumeration of *Listeria monocytogenes* and of *Listeria* spp.—Part 1: Detection Method.

[25] Scatassa M.L., Miraglia V., Carrozzo A., Ducato B., Lazzara F., Todaro M., Mancuso I. (2012). Determination of Chemical Parameters by Means of NIR Technology (FoodScan™ Dairy Analyser) in “Caciocavallo Palermitano” Cheese Samples. Proceedings of the XIV Congresso Nazionale S.I.Di.L.V..

[26] (2016). Sensory Analysis—Methodology—General Guidance for Establishing a Sensory Profile.

[27] (2007). Sensory Analysis—General Guidance for the Design of Test Rooms.

[28] Caccamo M., Pasta C., Petriglieri R., Difalco A., Calandra Checco G.A., Farina G., Belvedere G., Marino G., Alcaine S.D., Caggia C. (2025). The affinage of cheese using artisanal beers from ricotta whey: A sustainable way to differentiate traditional cheeses. Appl. Sci..

[29] Ares G., Gámbaro A. (2007). Influence of gender, age and motives underlying food choice on perceived healthiness and willingness to try functional foods. Appetite.

[30] Grunert K.G., Hieke S., Wills J. (2014). Sustainability labels on food products: Consumer motivation, understanding and use. Food Policy.

[31] Cammarelle A., Viscecchia R., Bimbo F. (2021). Intention to Purchase Active and Intelligent Packaging to Reduce Household Food Waste: Evidence from Italian Consumers. Sustainability.

[32] Brown S.R.B., Forauer E.C., D’Amico D.J. (2018). Effect of modified atmosphere packaging on the growth of spoilage microorganisms and *Listeria monocytogenes* on fresh cheese. J. Dairy Sci..

[33] Burall L.S., Laksanalamai P., Datta A.R. (2012). *Listeria monocytogenes* mutants with altered growth phenotypes at refrigeration temperature and high salt concentrations. Appl. Environ. Microbiol..

[34] Roberts B.N., Chakravarty D., Gardner J.C., Ricke S.C., Donaldson J.R. (2020). *Listeria monocytogenes* response to anaerobic environments. Pathogens.

[35] Radford D., Guild B., Strange P., Ahmed R., Lim L.T., Balamurugan S. (2017). Characterization of antimicrobial properties of *Salmonella* phage Felix O1 and *Listeria* phage A511 embedded in xanthan coatings on poly(lactic acid) films. Food Microbiol..

[36] European Commission (2005). Commission Regulation (EC) No 2073/2005 of 15 November 2005 on microbiological criteria for foodstuffs. Off. J. Eur. Union.

[37] Iacumin L., Manzano M., Comi G. (2016). Phage inactivation of *Listeria monocytogenes* on San Daniele dry-cured ham and elimination of biofilms from equipment and working environments. Microorganisms.

[38] Franceschi P., Formaggioni P., Brasca M., Natrella G., Faccia M., Malacarne M., Summer A. (2023). Fatty Acids Composition and Lipolysis of Parmigiano Reggiano PDO Cheese: Effect of the Milk Cooling Temperature at the Farm. Anim. Biosci..

[39] Gobbetti M., Neviani E., Fox P. (2018). The Cheeses of Italy: Science and Technology.

[40] McSweeney P.L.H., Sousa M.J. (2000). Biochemical pathways for the production of flavour compounds in cheeses during ripening: A review. Lait.

[41] Sulejmani E., Hayaloglu A.A. (2020). Influence of starter culture on nitrogen fraction and volatile compounds in beaten cow’s milk cheese. J. Food Process. Preserv..

[42] Wolf I.V., Perotti M.C., Zalazar C.A. (2011). Composition and volatile profiles of commercial Argentinean blue cheeses. J. Sci. Food Agric..

[43] Pinho O., Pérès C., Ferreira I.M.P.L.V.O. (2003). Solid-phase microextraction of volatile compounds in “Terrincho” ewe cheese: Comparison of different fibers. J. Chromatogr. A.

[44] Ziino M., Condurso C., Romeo V., Giuffrida D., Verzera A. (2005). Characterization of “Provola dei Nebrodi”, a typical Sicilian cheese, by volatiles analysis using SPME-GC/MS. Int. Dairy J..

[45] Erkaya-Kotan T., Hayaloglu A.A. (2024). Volatilome Profile (HS-SPME/GC–MS) and Proteolysis in Beyaz Peynir (White-Brined Cheese) Made Using Different Probiotic Adjunct Cultures and Ripening under Brine or Vacuum Package Systems, and Chemometric Analysis. Int. Dairy J..

[46] Kontogianni V.G., Kosma I., Mataragas M., Pappa E., Badeka A.V., Bosnea L. (2023). Innovative intelligent cheese packaging with whey protein-based edible films containing Spirulina. Sustainability.

[47] Chirilli C., Torri L. (2023). Effect of Biobased Cling Films on Cheese Quality: Color and Aroma Analysis for Sustainable Food Packaging. Foods.

[48] Busetta G., Garofalo G., Barbera M., Di Trana A., Claps S., Lovallo C., Franciosi E., Gaglio R., Settanni L. (2023). Metagenomic, Microbiological, Chemical and Sensory Profiling of Caciocavallo Podolico Lucano Cheese. Food Res. Int..

[49] Braghieri A., Girolami A., Riviezzi A.M., Piazzolla N., Napolitano F. (2014). Liking of traditional cheese and consumer willingness to pay. Italian J. Anim. Sci..

[50] Testa R., Schifani G., Migliore G. (2021). Understanding consumers’ convenience orientation: An exploratory study of fresh-cut fruit in Italy. Sustainability.

[51] Laureati M., De Boni A., Saba A., Lamy E., Minervini F., Delgado A.M., Sinesio F. (2024). Determinants of consumers’ acceptance and adoption of novel food in view of more resilient and sustainable food systems in the EU: A systematic literature review. Foods.

[52] Frewer L.J. (2017). Consumer acceptance and rejection of emerging agrifood technologies and their applications. Eur. Rev. Agric. Econ..

[53] Gagliardi F., Brogi L., Betti G., Riccaboni A., Tozzi C. (2025). Italian consumer willingness to pay for agri-food sustainable certification labels: The role of sociodemographic factors. Sustainability.

[54] Grunert K.G., Sonntag W.I., Glanz-Chanos V., Forum S. (2018). Consumer interest in environmental impact, safety, health and animal welfare aspects of modern pig production: Results of a cross-national choice experiment. Meat Sci..

[55] Moatsou G. (2024). Emerging Technologies for Improving Properties, Shelf Life, and Analysis of Dairy Products. Foods.

[56] Ghazanfari S., Firoozzare A., Covino D., Boccia F., Palmieri N. (2024). Exploring Factors Influencing Consumers’ Willingness to Pay Healthy-Labeled Foods at a Premium Price. Sustainability.

